# Environmental Temperature in Thermal Comfort Under Different Virtual Tourism Activity Intensities: Based on Microclimate Simulation Experiment

**DOI:** 10.3389/fnins.2021.762322

**Published:** 2022-01-31

**Authors:** Linqiang Wang, Jiahui Wang, Xiaoting Huang, Honglei Chi

**Affiliations:** ^1^School of Management, Shandong University, Jinan, China; ^2^Jinan Lixia Holding Group Co., Ltd., Jinan, China

**Keywords:** virtual tourism, thermal comfort, activity intensity, experimental research, semi-structural interview

## Abstract

Although COVID-19 lockdowns and travel regulations have restricted the spatial area for human activities, tourists can still use virtual devices and applications for travel purposes. This study aimed to explore the thermal comfort and satisfaction of tourists under various tourist activity intensities, using experimental and semi-structured interview methods, combined with microclimate simulation experiments and electrocardiogram data to monitor physiological indicators. The results showed that: (1) The thermal comfort of virtual tourists had a significant correlation with the environmental temperature. (2) The thermal comfort of virtual tourists differed under various activity intensities. The virtual tourism activity intensity moderated the relation between environmental temperature and tourists’ thermal comfort. (3) In the state of exercise (slow walking, fast walking), the environmental temperature affected tourists’ physiological indicators. (4) Virtual tourism that integrates realistic visual, audio, and tactile sensations can improve tourists’ perception and satisfaction. The results provide a new perspective for the study of the virtual tourism experience and thermal comfort. In addition, it provides theoretical and practical support for the development of virtual tourism scenes in the environmental temperature context.

## Introduction

The COVID-19 pandemic has dramatically reduced the number of tourists worldwide. In 2020, the numbers of international arrivals fell by 74% from the previous year with financial losses of 1.3 trillion US dollars, becoming “the worst in the history of tourism years”. In the post-lockdown period of the pandemic, although the tourism industry has shown the ability to quickly recover and return to normality, the uncertainty shock of pandemics still poses danger to the industry. The tourism industry needs transformation and upgrading. Due to improvements in technology, virtual reality (VR) has addressed the limitations posed by simulating the environment through pictures and videos (Carmigniani et al., 2011; Choi et al., 2015; Bogicevic et al., 2019), providing a better “immersion” experience (Bohil et al., 2011). Virtual tourism has sparked a surge of related products and services (Burdea and Coiffet, 2003; Serino et al., 2014). Virtual tourism not only solves the problem of boredom at home during the pandemic but also meets the huge social and market needs of the Internet era.

The following questions can be posed: Is virtual tourism just for tourists to obtain audiovisual experiences through technical means? How can the perfect virtual tourism experience be obtained? The experience needs to be integrated with microclimate conditions to enhance tourists’ sense of presence and activity comfort, enabling them to have an immersive tourism experience (Zheng et al., 2010). The biggest advantage of virtual tourism is the high degree of tourist participation and interaction with the virtual travel experience (Huang et al., 2020). However, its realization depends on the tourists’ Internet-connected devices; it cannot replace an on-site tour, let alone replicate the various atmospheres and sense of fun (Minderer et al., 2016). Therefore, it is essential to strengthen the sense of experience and simulation of virtual tourism (Riva et al., 2011), especially to meet the need for tourists to have an immersive experience based on the development of temperature-related environmental virtual tourism scenes.

Climate is one of the biggest factors affecting human comfort (Nicol and Humphreys, 1973; de Dear and Brager, 1998), and temperature has been utilized as an important tourism resource (Clements-Croome, 2013). Some tourism activities developed according to the climate have become popular choices for tourists, for example, migratory bird tourism, residence tourism, and retirement tourism (Hao et al., 2020). To date, few studies in the literature have researched the climate comfort of tourism activities, let alone the climate comfort of virtual tourism activities. Developing climate and temperature-related virtual tourism products without theoretical guidance means that tourists will not have pleasant and high-quality travel experiences in their virtual tourism activity (Bell et al., 2003). Therefore, it is necessary to explore virtual tourists’ thermal comfort and their satisfaction with virtual travel experiences.

The study used both qualitative and quantitative methods, conducting a 3 × 3 experiment and semi-structured interview to explore two important areas: (1) A microclimate simulation experiment was used to simulate the environmental temperature under different types of virtual tourism activity intensity; virtual tourists’ thermal comfort were analyzed by quantitative research. (2) Tourists’ satisfaction was further explored under various virtual tourism activity intensities through semi-structured interviews, revealing the new characteristics of virtual tourism thermal comfort, thus promoting the sustainable development of the virtual tourism industry.

## Literature Review

### Virtual Tourism and Virtual Tourism Experience

Virtual tourism emerged in the 1990s; Perry and Williams (1995) first defined virtual tourism as a new format resulting from the combination of VR technology and tourism. Virtual tourism refers to the use of computer simulation technology, VR technology, and augmented reality (AR) technology to dynamically present real or non-existent tourism landscapes to tourists (Heeter, 2000; Wrzesien et al., 2013). Tourists can obtain an immersive travel experience without leaving home (Lin et al., 2020). With the continuous development of GIS (Geographic Information System), three-dimensional visualization, virtual reality, 3D Internet, and other technologies (Wrzesien et al., 2013; Brown and Green, 2016), virtual tourism can not only use computers to process graphics, images, videos, sound, and animation, but also present three-dimensional entities and the three-dimensional environment in a virtual form, realizing interactive three-dimensional animation and dynamic simulation (Choi et al., 2015; Botella et al., 2017; Loureiro et al., 2020).

On-site tourism brings tourists on site, giving them a real experience, while virtual tourism is a mirror-image simulation of a travel experience, which is the core of the entire virtual tourism activity (Williams and Hobson, 1995). Xu et al. (2001) compared the interactive mechanisms of virtual tourism and real tourism, finding a strong correlation between acquisition path and perception effect. The virtual tourism experience is not designed simply to meet the needs of audiovisual senses; its ultimate goal is to provide a full sensory immersive experience that integrates interaction, immersion, and artistic conception (Kaptelinin and Nardi, 2006; Jung et al., 2016; Calogiuri et al., 2018). In the future, virtual tourism will not be limited to fixed public places such as 9D virtual reality experience halls, VR experience centers, or VR theme parks. It could even control audiovisual systems, lighting systems, door and window systems, and other indoor smart home control systems to create private smart virtual tourism projects (Liu, 2020).

### Virtual Tourism Activity Types

In a virtual tourism experience, tourists enter a set virtual tourism scene through VR technology and use a variety of interactive devices such as helmets, data gloves, or sensory feedback devices to meet the needs of the scene and tasks and to control the environment (Tatzgern et al., 2015). Virtual tourists can “go through” to the scenic spots, communicate with other tourists, take part in exercise such as hiking or adventure, and even manipulate “objects” (such as plants and animals) in the virtual scenes (Martinez-Grana et al., 2013). According to the different interactions between tourists, there are four types of virtual tourism: desktop virtual tourism, cabin-style virtual tourism, immersive virtual tourism, and naked-eye 3D virtual tourism (García-Crespo et al., 2016; Myung et al., 2020).

In desktop virtual tourism, tourists experience virtual world landscapes through computer monitors or mobile terminal devices. These landscapes are mainly rendered by 360° three-dimensional real-world technology (Liu and Deng, 2017), which can be operated in a 360° direction and watched on any terminal (Choi et al., 2015). Chinese famous tourist attractions and cities can be browsed from all angles on virtual tourism websites such as “China Panorama Network” and “Panorama Virtual Travel Network.” In cabin-style virtual tourism, tourists are sited in a special cabin equipped with a screen that can watch the virtual world. The virtual world can be observed from different angles by rotating the cabin. The users don’t need to wear other special devices to engage in interactive activities in the virtual world, which they can do so in a burden-free manner (Huang et al., 2020). In immersive virtual tourism, tourists need to be equipped with helmet-mounted displays or wrap-around monitors to experience the virtual travel world (Burdea et al., 1996; Mattila et al., 2020) and take part in various virtual tourism activities. The scene is stronger and more realistic. In naked-eye 3D virtual tourism, AI, eye tracking, and 3D rendering is used to track the tourists’ eyeballs through an integrated camera. The system performs AI calculations based on the captured eye movements and dynamically presents realistic beaches, forests and fields, and other landscapes, aiming to provide tourists with an immersive naked-eye 3D viewing experience (Wrzesien et al., 2013).

### Tourism Thermal Comfort

Changes in climatic conditions such as temperature and humidity comprehensively affect the comfort of the human body, which in turn has, to varying degrees, health and physiological effects on the human body. Therefore, climate comfort is a very important factor in human activities. Climate comfort evaluation models and indicators have been extensively studied in the fields of architectural design (Hellwig, 2015), urban planning (Parkinson and de Dear, 2015), human health, and tourism development (Zhou et al., 2014). Tourism climate was first introduced by Hibbs (1966), who pointed out that tourism climate is a tourism resource with beneficial or adverse effects within a certain time and space, which can be used for tourism development and can be quantitatively evaluated. Tourism climate has gradually become a new research field; some indicators specifically aimed at measuring tourism climate comfort have been proposed, such as CI (Climatic-tourist Index), TCI (Tourism Climate Index), and CIT (Climate Index for Tourism) (Fang and Yin, 2015; Nalau et al., 2017). Climate comfort has gradually become an important determinant in the tourism decision-making process (Brager and de Dear, 1998).

Temperature is a key factor that impacts human comfort and is an important indicator for climate comfort. Research on thermal comfort includes significant guidance on helping tourists to make travel decisions and select tourist destinations (Mieczkowski, 2010). The existing research on thermal comfort mainly focuses on the influence mechanism, and the temporal and spatial distribution of the thermal comfort zone. However, in thermal comfort evaluation research, the body temperature classification standard varies in different conditions and cannot be simply applied to other contexts. For example, the “pleasant climate” varies from region to region, according to the effective temperature; the “pleasant climate” ranges from 15–19°C in the United Kingdom, 20–26°C in Europe and Indonesia, 21–26°C in India, and 21–29°C in Malaysia (Lise and Tol, 2002; Hamilton, 2004). The thermal comfort of tourism not only affects the length of the comfortable period of tourism and the choice of tourist destination, but also affects the changes in tourism activities and the function of tourism resources (Lu et al., 2016).

### Hypotheses

**FIGURE 1:**
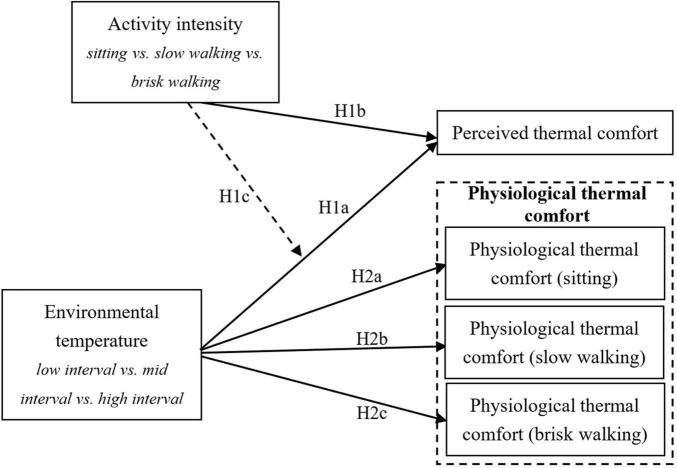
Conceptual model and hypotheses.

The study examined the effect of environmental temperature and virtual tourism intensity on tourists’ thermal comfort, and whether the environmental temperature has an impact on tourists’ physiological indicators under various virtual tourism intensities. The conceptual model is shown in Figure 1.

With the extensive development of indoor tourism activities such as museum tours, temperature has become the environmental factor that is the easiest to change. In outdoor tourism activities, the temperature of destinations can be changed by increasing vegetation coverage and water areas, providing tourists with a healthy and comfortable tourism environment. According to the human body thermal balance theory, there is heat exchange between the human body and the environment. When the heat generated by the human body is equal to the heat emitted, the human body is in a thermally comfortable state. Numerous studies have also shown that environmental temperature affects the thermal comfort of tourists (Schellen et al., 2013).

According to exercise physiology, metabolic rate is a representative indicator of human energy metabolism and has an important influence on thermal sensation and comfort. During exercise, the human body has a high metabolic rate, with the low thermal resistance of clothes, a large amount of perspiration, and shortness of breath. There is a significant difference in the thermal comfort perceived by the human body in the exercise state compared to the quiet sitting state (Ji et al., 2015). A recent study conducted by Zhang et al. (2020) found that under different weather conditions, exercise intensity has a significant impact on the thermal comfort of the human body. In addition, in different exercise states, the human body will reach a state of equilibrium through the contraction and relaxation of blood vessels, perspiration, and shivering. Compared with the sitting posture, the human body has a greater range of temperature acceptance and adaptation during exercise. People who are in a thermally comfortable state during exercise have lower skin temperature, higher sweating rate, and higher core body temperature (Revel and Arnesano, 2014). The physiological functions of the human body change greatly under different exercise levels; the acceptance and adaption range of temperature is not always the same, which affects the thermal comfort of the human body (Yin et al., 2017). Therefore, the study considered the effect of activity intensity which is closer to the actual virtual tourism activity and enhances the stimulating effect. Thus, it was meaningful to consider the influence of activity intensity that is close to the actual virtual tourism experience, as proposed in the following hypotheses:

*H1: Environmental temperature and activity intensity influence virtual tourists’ perceived thermal comfort.*

*H1a: Environmental temperature influences virtual tourists’ perceived thermal comfort.*

*H1b: Activity intensity influences virtual tourists’ perceived thermal comfort.*

*H1c: Activity intensity moderates the relationship between environmental temperature and virtual tourist’s perceived thermal comfort.*

According to tourism embodied theory, tourists’ experience is the result of the interaction of a series of related elements such as perception, the body, and the environment in the travel process. Thermal comfort during tourism activities is impacted by both physiological and psychological factors. Physiological indicators are important in studies of thermal comfort. Previous studies have shown that tourists, as warm-blooded mammals, can maintain a relatively constant body temperature when the environmental temperature changes. Under the control of the temperature regulation center (hypothalamus), the human body’s heat production and heat dissipation processes are changed by physiological adjustment responses such as blood flow to the skin, vasoconstriction, and sweating rate. In addition, the thermal environment can also cause changes in water and salt metabolism, the cardiovascular system, and the nervous system. Studies have indicated that the higher the environmental temperature, the greater the labor intensity and the faster the heart rate (Boo-Chai, 1978). Kanosue et al. (2002) argued that the more uncomfortable people are, the higher the blood oxygen-dependent level of the tonsils on the sides of the brain. A study conducted by Xiong et al. (2016) has shown that indicators such as serum IL-6, oral temperature, skin temperature, heart rate, and heart rate variability are sensitive to temperature changes; thus, these indicators can potentially reflect the effect of temperature on human health and thermal comfort. This study used the seven indicators of HR (heart rate), PR (pulse rate), SPO_2_ (blood oxygen), NIBP-Dia (diastolic blood pressure), NIBP-Sys (systolic blood pressure), NIBP-Mean (average blood pressure), and RR (respiratory rate) as proxies for the human physiological function change process, exploring how the environmental temperature influenced virtual tourists’ physiological data under different activity intensities. Based on previous research, this study posits the following hypothesis:

*H2: Under different tourism activity intensities, environmental temperature influences virtual tourists’ physiological thermal comfort.*

*H2a: In the state of stability (sitting), environmental temperature influences virtual tourists’ physiological indicators.*

*H2b: In the state of slow walking exercise, environmental temperature influences virtual tourists’ physiological indicators.*

*H2c: In the state of brisk walking exercise, environmental temperature influences virtual tourists’ physiological indicators.*

## Materials and Methods

### Experiment Instruments

**FIGURE 2:**
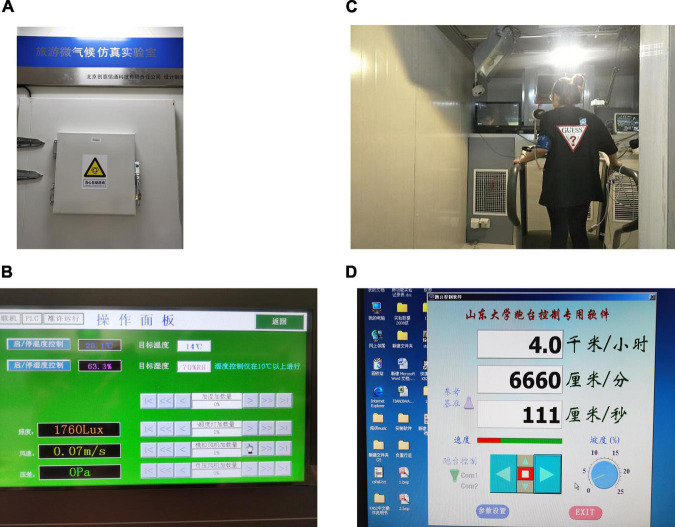
Experimental instruments. **(A)** The appearance of microclimate warehouse. **(B)** The operation panel of microclimate warehouse. **(C)** Running platform, LED screen, ECG measuring instrument. **(D)** Running platform control center.

The experiment was conducted in a tourism microclimate simulation laboratory in an eastern Chinese university. A window on the cabin door enabled the researcher to observe the experimental situation. There are temperature sensors, air humidifiers, LED lamps, and blowing ports in the cabin; thus, the temperature, humidity, wind speed, light, and other environmental factors can be controlled through the control panel outside the cabin. It can be heated and cooled in a short period, and the convenient operation makes it easy to simulate different virtual tourism environments in research on tourism comfort. The experimental instruments included a running platform, LED display screens (used to play documentaries of tourism scenes and to simulate the virtual tourism environment, which is the essential difference between this experiment and other thermal comfort experiments), and an electrocardiograph (testing the subjects’ physiological indicators). The experimental instruments and pictures of them are shown in Table 1 and Figure 2.

**TABLE 1:** Experimental instruments.

Instruments	Simulation/test content	Units
Microclimate warehouse	Air temperature	°C
	Relative humidity	%
	Wind speed	m/s
	Illuminance	Lux
ECG measuring instrument	PR (pulse rate)	Bmp
	HR (heart rate)	Bmp
	SpO_2_ (pulse oxygen saturation)	%
	NIBP (blood pressure)	mmHg
	RR (respiratory rate)	rpm
Running platform	Activity speed	m/s

### Virtual Tourism Scenarios

Travel is the process of interaction between tourists and the travel time and space environment. Tourism experimental research starts with the establishment of tourism scenes. This is also the biggest difference between tourism experimental research and exercise physiology experiments in other disciplines. In the experiment, climate environment parameters consistent with the outdoor environment were set and controlled by the microclimate cabin, including temperature, humidity, solar illumination, and wind speed. The LED display screen was used to play tourism documentaries to simulate different travel scenes, and the speed and slope were adjusted through the treadmill to simulate the three activity forms of sitting, walking slowly, and walking quickly, thus simulating different tourism activities in the virtual tourism situation. According to previous studies on tourism climate comfort (Qian and Ye, 1996; Dahai and Rong, 2000), the optimum value of humidity is 70%, wind speed is 2 m/s, and illuminance is 4200 lux.

### Experimental Design

The study examined whether environmental temperature and activity intensity can influence tourists’ thermal comfort. To simulate the climate environment in different activity intensities and obtain both physiological and psychological data, each respondent participated in two factorial (temperature and activity intensity) repeated experiments. This also helped to avoid the errors caused by individual differences. Therefore, this study employed a 3 (environmental temperature: low interval, mid-interval, and high interval)°× 3 (activity intensities: sitting, slow walking, and brisk walking) between-within subjects experimental design.

Based on the suggestion that the environment has an impact on the human body (Büttner, 1938), an increasing number of research studies have used the human body thermal balance model in thermal comfort assessment. The change of the human body’s thermal balance is an important factor that influences human physiological parameters. The human body thermal balance equation is:


$${\text{Q}}_{m}={\text{Q}}_{C}+{\text{Q}}_{r}+{\text{Q}}_{\mathrm{res}}+{\text{Q}}_{\mathrm{es}}+{\text{Q}}_{\mathrm{st}}$$

*Q*_*m*_ is the heat production of human body metabolism, unit: W/m^2^; *Q*_*c*_ is the heat that the human body emits to the surrounding environment through convection, unit: W/m^2^; *Q*_*r*_ is the heat emitted from the human body surface to the surrounding environment through radiation, unit: W/m^2^; *Q*_*res*_ is the heat taken away by exhaled water vapor, unit: W/m^2^; *Q*_*es*_ is the heat taken away by sweat evaporation, unit: W/m^2^; *Q*_*st*_ is the heat storage of the human body, unit: W/m^2^.

When *Q*_*st*_ = 0, the human body is in a state of thermal equilibrium. In a low-temperature environment, the heat storage of the human body declines and leads to *Q*_*st*_<0, and people feel cold and uncomfortable. In a high-temperature environment, heat accumulates in the body, resulting in *Q*_*st*_ > 0, and people feel hot and uncomfortable. Fanger’s (1970) equation is established under the conditions of normal temperature and low activity level, and it is difficult to accurately predict the thermal comfort of the human body in the state of exercise. Thus, it is more suitable to conduct tourist exercise studies from the perspectives of exercise physiology and environmental psychology (Merla et al., 2010). Under different levels of exercise, the human body maintains a balanced state through the contraction and dilation of blood vessels, perspiration, and shivering. In addition, the body’s physiological functions and comfort change significantly under various activity intensities (Ji et al., 2015).

The physiological equivalent temperature index (PET index) refers to the climate temperature in which the human skin temperature and in-body temperature reach the same thermal state in a certain indoor or outdoor environment. Qiu et al. (2005) explored the human sweat regulation mechanism under different temperature circumstances and different exercise intensities in human experiments, revealing the relationship between environmental temperature and different types of human activity intensities. Considering the health of respondents, the study adopted three mild temperature intervals in the PET index: 13.1–18.0°C (slightly cool, light cold pressure), 18.1–23.0°C (comfortable, no heat pressure), and 23.1–29.0°C (slightly warm, light heat pressure). These three temperature intervals have been used in application examples of PET indicators (Höppe, 1999; Matzarakis et al., 1999; Shang et al., 2020), and each large temperature interval was subdivided into three small ranges to obtain a more accurate thermal comfort result. With a slight modification to the existing research (Qiu et al., 2005), the current study used the following common virtual tourism activity intensities: sitting, slow walking, and brisk walking (Table 2).

**TABLE 2:** Examples of tourism scenarios under different activity intensities.

Activity intensities of the experimental design	Common scenarios in virtual tourism activities	Literature sources
Sitting	360° 3D panoramic desktop display browsing of tourism cities, tourism scenes, and virtual attractions.	Griffon et al., 2011, Chen, 2013
	Preview the hotel accommodation conditions by moving the screen and pointer.	Lee and Oh, 2007
Slow walking	A panoramic walking tour of VR theme park scenic spots through helmet-mounted displays or omni-directional monitors.	Lin and Zhang, 2017; Yu, 2017; Sutherland, 1968
	Watch the set underwater world, volcanoes, etc., through the mobile screen in the special cabin.	Huang et al., 2020
Brisk walking	Interactive sports such as running or climbing in naked-eye 3D virtual tourism scenes.	Lewis and Rosie, 2012
	Perform virtual game operations as a pilot, astronaut, etc., on the advanced simulator.	Jiang et al., 2007

The specific experimental requirements are casual and comfortable posture when sitting quietly; the pace of slow walking is 0.7 m/s, and the pace of brisk walking for men is 1.3 m/s and for women is 1.1 m/s (Delp et al., 2007). The nine temperature sub-intervals and three activity intensities constitute 27 experimental conditions (Table 3). The physiological data were collected using electrocardiographs while the experiment was being conducted.

**TABLE 3:** Experimental design.

Activity intensity Temperature (°C)		Sitting	Slow walking	Brisk walking
A: 13.1–18.0	A1: 13.1–14.7	A11	A12	A13
	A2: 14.8–16.3	A21	A22	A23
	A3: 16.4–18.0	A31	A32	A33
B: 18.1–23.0	B1: 18.1–19.7	B11	B12	B13
	B2: 19.8–21.3	B21	B22	B23
	B3: 21.4–23.0	B31	B32	B33
C: 23.1–29.0	C1: 23.1–25.0	C11	C12	C13
	C2: 25.0–27.0	C21	C22	C23
	C3: 27.1–29.0	C31	C32	C33

*Controlled conditions: relative humidity (70%), wind speed (2 m/s), illuminance (4200 lux).*

### Data Collection and Sample

The experiment was conducted from February 2019 to August 2020. Sixty respondents were recruited through the TBL WeChat public platform; only respondents older than 18 years old with no history of heart disease or other health problems were eligible to participate in the experiment. The sample comprised 30 males and 30 females. The majority of respondents were between 18 and 30 years old; the average age was 21.72 years old. BMI was an important criterion for selecting respondents; the BMI of respondents was between 20 and 23, with an average of 21.26.

All the respondents had to uniformly wear short-sleeved T-shirts and trousers to mitigate the effect of the thermal-resistant clothes. At the same time, respondents needed to have a good night’s sleep and not to exercise strenuously or diet before the experiment.

The respondents were randomly divided into three groups, each of which contained ten males and ten females. In the three activity states, the first group experienced the temperature range A1+B1+C1, the second group experienced the temperature range A2+B2+C2, while the third group experienced the temperature range A3+B3+C3. This helped to eliminate measurement errors caused by individual differences. Each respondent carried out nine sets of the experiment, and each experiment lasted 10 min. To avoid the influence of the previous experiment, the subject returned to their resting state after each experiment.

### Measuring Tourist Response Variables

Environmental climatic data and activity intensity are posited as antecedents. Climatic variables such as humidity, wind speed, and illumination were controlled within a comfortable range. Temperature was the only climatic variable in the study and was divided into nine intervals. Activity intensity was divided into three states: sitting quietly, walking slowly, and brisk walking.

The physiological indicators and perceived thermal comfort were set as outcomes. Considering the mobile nature of tourism, the experiment mainly collected data on seven basic indicators that can reflect the changing process of human physiological functions during exercise, including HR, PR, SPO_2_, NIBP-Dia, NIBP-Sys, NIBP-Mean, and RR.

Thermal comfort is the subjective feelings of virtual tourists. Psychophysiologists use numerical scales to quantify these feelings. In order to express the relationship between scores and comfort intuitively, the study adopted the human thermal comfort scale (four-level index, 0 = “very uncomfortable,” 1 = “uncomfortable,” 2 = “a little comfortable,” 3 = “comfortable”) (Deb and Ramachandraiah, 2011).

### Interview Outline

To gain a comprehensive understanding of tourists’ thermal comfort under different virtual tourism intensities and complement the quantitative research, qualitative data collection was also conducted. Semi-structured interviews were conducted with the 60 subjects who participated in the experiment; they had a good understanding of the virtual tourism experience. An in-depth interview lasting around 20 min was conducted with each of these subjects. The interview outline mainly included travel preferences, feelings about the experiment, suggestions, and open questions. The interviews began in May 2019 and ended in October 2020.

At the beginning of the interview, each interviewee was assured that the recording would only be used for the purpose of this research. After obtaining their consent, the conversation was recorded using recording device. During the interviews, researcher briefly recorded the respondents’ answers, reactions, and problems that arose, and adjusted the interview strategy according to the actual situation. After the interview, referring to the interview notes, researchers transcribed the interview content verbatim into a recording script, listened to the recording repeatedly and proofread it, finally producing an interview transcript for subsequent analysis.

The study employed a variety of measures to ensure the validity and reliability of the interview data. First, three test interviews were conducted to help the researcher gain skills prior to the formal interview, and some questions were modified based on participants’ feedback. Second, to build rapport and establish comfortable interactions between the researcher and interviewees, measures were taken in advance of the interview and also during the interview itself. A summary of the project and interview outline were sent to the participants before the interview either via e-mail or text message, and the interview time was mutually agreed upon. During the interview, the researcher aimed to create a relaxed atmosphere and adjust the opening question according to the actual situation, recording the important content at the same time. Third, if the content of the interview data was found to be unclear, the researchers would conduct a second interview via e-mail or telephone call to ensure that the qualitative data truly reflected the participants’ experiences. Finally, a panel of two researchers combined to analyze and theoretically label participants’ responses: one researcher conducted the initial analysis, while another researcher who was well acquainted with the study checked this independently.

The socio-demographic profile of the respondents is presented in Table 4.

**TABLE 4:** Socio-demographic profile of the interviewees.

Demographic		Frequency	Percentage
Gender	Male	30	50.00
	Female	30	50.00
Age	<18	13	21.67
	18–30	32	53.33
	30–40	13	21.67
	>40	2	3.33
Education	Bachelor’s degree or under	25	41.67
	Master’s degree	24	40.00
	Doctor’s degree	11	18.33
Climate zone of residential place	Tropical	11	18.33
	Subtropical	21	35.00
	Temperate	28	46.67
Virtual tourism experience times	0	6	10.00
	1–5	30	50.00
	6–10	18	30.00
	More than 10	6	10.00
			

### Experiment Steps

The experiment steps were as follows.

Step 1: A health survey of registered respondents was conducted to ensure that they did not have a history of heart disease or other health problems. Respondents were reminded to prepare clothing to wear for the experiment. After the respondents arrived in the laboratory, they were informed of the specific content of the experiment, precautions, and emergency response measures. Respondents were asked to sign the experimental informed consent form and to provide basic information and complete a pre-test questionnaire. The subjects’ pulse and blood pressure indicators needed to be within the normal range; if not, they had to sit still until their heart rate and pulse returned to a resting state.

Step 2: A virtual tourism microclimate experimental scenario was set up. Tourism is a process of interaction between tourists and the tourism environment; the simulation of the tourism situation is the essential difference between this experiment and other thermal comfort experiments. In the experiment, the microclimate cabin set and controlled the simulated climate environment consistent with the outdoor environment, including temperature, humidity, solar illumination, and wind speed. The LED display screen was used to play tourism documentaries, while the speed and slope angle were adjusted through the treadmill to simulate three activity intensities (sitting, slow walking, and brisk walking). This enabled the simulation of different tourism activities in virtual tourism situations.

Step 3: Every respondent took off metal accessories, wore an electrocardiograph, entered the tourism microclimate cabin, and completed nine sets of virtual tourism experiments. After respondents finished each experiment, they were asked to evaluate their perceived thermal comfort using a four-point scale ranging from 0 to 3. Before starting the next set of experiments, respondents had to wait until their pulse, heart rate, and other physiological indicators returned to a resting state. Researchers also adjusted the climate parameters in the microclimate cabin to stabilize within the proper range.

Step 4: Semi-structured interviews were conducted after the respondents finished all experiments, which helped to fully understand their thermal comfort experience under different virtual tourism activity intensities. All the conversations were recorded and summarized.

## Experiment Analysis

### Relation With Perceived Thermal Comfort

The study used SPSS 24.0 software to analyze the interactive relationships between environmental temperature, activity intensities, perceived thermal comfort, and physiological thermal comfort.

It was proposed that when tourists experience virtual tourism activity, they are influenced by environmental temperature and activity intensity (sitting, slow walking, brisk walking), which affects their perceived thermal comfort (H1). The ANOVA results (Table 5) provided partial support for H1 as environmental temperature showed significant differences regarding perceived thermal comfort. The *P*-value is lower than 0.05, revealing significant differences between the low, mid, and high intervals. Thus, H1a was supported. The *P*-value of activity intensity is greater than 0.05, revealing no significant differences between sitting, slow walking, and brisk walking. Therefore, H1b was rejected. H1c predicted that there will be a significant interaction between environmental temperature and activity intensities. The ANOVA also tested the interaction between these two variables; the results (Table 5) showed that the *P*-value of interaction is lower than 0.05, which indicated that activity intensity had a moderate effect on the relationship between environmental temperature and perceived thermal comfort. Therefore, H1c was supported.

**TABLE 5:** ANOVA: environmental temperature, activity intensity, and perceived thermal comfort.

	Temperature	Activity intensity	Temperature*activity intensity
*F*	25.74***	1.91	6.96***
*P*	0.000	0.126	0.000

*p < 0.05; **p < 0.01; ***p < 0.001.

In the state of sitting, the lowest value of perceived thermal comfort was between 13.1 and 16.3°C. Tourists’ sensations changed from uncomfortable to a little comfortable in the interval 16.4–18.0°C; thus, the inflection point was in this interval. The comfort value reaches the highest point in the interval 21.4–25.0°C. When the temperature was higher than 25°C, the comfort value dropped slightly. In summary, in the sitting state, the majority of virtual tourists had a good acceptance of the temperature at 16.4–29.0°C. In the interval 13.1–16.3°C, the temperature was cold and the experience was relatively poor for tourists.

In the state of slow walking, the lowest value of perceived thermal comfort was again between 13.1 and 16.3°C, but higher than the counterpart in the state of sitting quietly. The turning point was also in the interval 16.4–18.0°C. In this state, no virtual tourists gave the highest scores for perceived thermal comfort, which means that there was no comfortable temperature range when tourists were walking slowly. In the interval 19.8–21.3°C, perceived thermal comfort reached the highest value. When the temperature was higher than 21.3°C, the perceived thermal comfort of virtual tourists dropped a little. Overall, in the interval 16.4–29°C, all virtual tourists accepted the temperature well, while tourists felt cold and found the experience poor in the interval 13.1–16.3°C.

In the state of brisk walking, the perceived thermal comfort values between 13.7 and 27.0°C were all greater than 2, indicating that most tourists felt comfortable within this temperature range. When the temperature was higher than 27.0°C, the comfort level dropped quickly and tourists felt a little hot. In summary, in the state of brisk walking, no temperature range made all respondents feel comfortable. Compared with other intervals, tourists felt more comfortable between 19.8 and 21.3°C.

**FIGURE 3:**
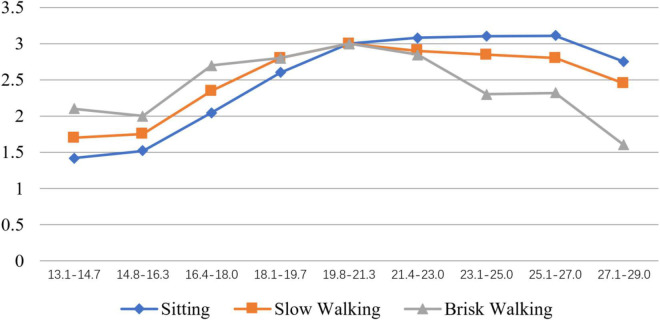
Virtual tourist’s comfort level-temperature under different activity intensity.

In summary, as the temperature increased, the virtual tourists’ comfort level first increased and then decreased; under different activity intensities, the subjects’ comfort levels also differed within the same temperature interval. Figure 3 shows the detailed relations among perceived thermal comfort, environmental temperature, and activity intensity in the virtual tourism context.

According to Figure 3, the perceived thermal comfort under different virtual tourism activity intensities all increased first and then decreased. In the lower temperature range, the comfort level gradually increased with the increase in temperature, and in the middle-temperature range, it reached a higher comfort level and was relatively stable. In the higher temperature range, the comfort level gradually decreased as the temperature increased. The overall change pattern centered on the interval of 19.8–21.3°C: the higher the activity intensity, the higher the comfort level. When the temperature was higher than 19.8–21.3°C, the higher the activity intensity, the lower the comfort level. Compared with slow walking and sitting quietly, the respondents’ comfort value during brisk walking decreased at a faster rate.

**FIGURE 4:**
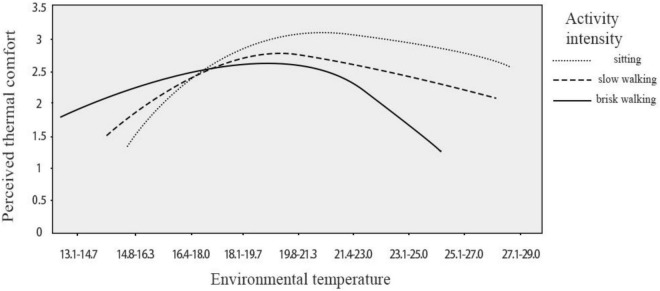
Moderating effect of activity intensity on the relationship between environmental temperature and perceived thermal comfort.

Therefore, the results also showed that the intensity of virtual tourism activities cannot directly affect the perceived thermal comfort of tourists. However, activity intensity can be used as a moderator to adjust the relationship between environmental temperature and perceived thermal comfort. Thus, H1a and H1c were supported, while H1b was rejected. Figure 4 illustrates the moderating effect of activity intensity on the relationship between environmental temperature and perceived thermal comfort.

### Relation With Physiological Thermal Comfort

The respondents’ resting physiological indicators were tested and recorded before the experiment. As previously mentioned, 60 subjects were randomly divided into three groups: the subjects in group 1 experienced the temperature range A1+B1+C1 in the three activity states, group 2 experienced the temperature range A2+B2+C2 in the three activity states, while group 3 experienced the temperature range A3+B3+C3 in the three activity states. The ANOVA revealed significant differences in natural blood pressure between group 1, group 2, and group 3 (Table 6). It cannot be concluded that the difference was caused by the change in experimental factors, so the blood pressure-related indicators were excluded in the further analysis. The *P*-values corresponding to HR, PR, SPO_2_, and RR are all greater than 0.05, indicating that there is no significant difference in the physiological indicators of the three groups in the natural state, meaning that these indicators could be included in the further analysis (Table 6).

**TABLE 6:** ANOVA: The resting physiological indexes in the pre-test.

Groups	HR	PR	SPO_2_	NIBP-Dia	NIBP-Sys	NIBP-Mean	RR
Group 1	77.88	75.83	98.00	107.94	68.06	76.24	17.24
Group 2	78.02	76.00	98.15	110.50	72.75	83.95	16.55
Group 3	78.00	76.15	98.24	113.29	74.53	83.65	16.82
*F*	1.381	1.824	0.849	7.874***	33.081***	51.089***	2.004
*P*	0.219	0.191	0.428	0.000	0.000	0.000	0.136

*p < 0.05, **p < 0.01, ***p < 0.001.

A homogeneity test was conducted before the ANCOVA (Table 7). The results showed that: in the state of sitting quietly, only the RR passed the homogeneity test (*F* = 2.406, *P* = 0.123); thus, RR and temperature were included in the covariance analysis. In the state of slow walking, PR (*F* = 0.213, *P* = 0.657), SPO_2_ (*F* = 0.168, *P* = 0.683), and RR (*F* = 1.879, *P* = 0.172) passed the homogeneity test; thus, PR, SPO_2_, RR, and temperature were included in the covariance analysis. In the state of brisk walking, SPO_2_ (*F* = 0.512, *P* = 0.475) and RR (*F* = 0.217, *P* = 0.642) passed the homogeneity test; thus SPO_2_, RR, and temperature were included in the covariance analysis.

**TABLE 7:** Homogeneity test of interaction under different activity intensities.

Activity intensity	Statistics	*HR*_resting*temperature_	*PR*_resting*temperature_	*SPO*_2_ _resting*temperature_	*RR*_resting*temperature_
Sitting	*F*	80.13***	254.96***	629.30***	2.41
	*P*	0.000	0.000	0.000	0.123
Slow Walking	*F*	54.73***	0.21	0.17	1.88
	*P*	0.000	0.657	0.683	0.172
Brisk walking	*F*	67.50***	11.28***	0.51	0.22
	*P*	0.000	0.001	0.475	0.642

*p < 0.05, **p < 0.01, ***p < 0.001.

The ANCOVA revealed that in the state of sitting, temperature did not have a significant effect on the virtual tourists’ physiological indicators (Table 8). In the state of sitting, the temperature does not significantly affect RR, due to the *P*-value being greater than 0.05. In the state of slow walking (*F* = 3.337, *P* = 0.001) and brisk walking (*F* = 2.390, *P* = 0.018), the temperature has a significant effect on tourists’ SPO_2_. In addition, in the state of slow walking, the temperature has a significant effect on PR (*F* = 2.272, *P* = 0.025). Therefore, in the stable state, the environmental temperature did not influence virtual tourists’ physiological indicators. In the state of exercise (slow walking, brisk walking), the environmental temperature influenced virtual tourists’ physiological indicators. Thus, H2 was partially supported, H2a was rejected, while H2b was supported.

**TABLE 8:** Results of the analysis of covariance.

Activity intensity	Statistics	*HR*	*PR*	*SPO*_2_	*RR*
Sitting	*F*	–	–	–	0.120
	*P*	–	–	–	0.998
Slow walking	*F*	–	2.272*	3.337**	0.16
	*P*	–	0.025	0.001	0.996
Brisk walking	*F*	–	–	2.390*	0.59
	*P*	–	–	0.018	0.787

*p < 0.05, **p < 0.01, ***p < 0.001.

In any state, the environmental temperature did not affect the RR significantly. The reason for this may be that temperature mainly affects the RR by affecting the activity of enzymes related to respiration. Due to the internal temperature regulation mechanism of the human body, the short experiment time and non-extreme environmental temperature, the subject can maintain a body temperature that fluctuates within the normal range. Therefore, in this experiment, the subjects’ respiratory enzyme activity did not change greatly; the environmental temperature had no significant effect on the RR.

## Interview Analysis

To supplement the quantitative study, we conducted qualitative research after the experiment to help understand respondents’ views with regard to their perceptions of virtual tourism and thermal comfort. The interview process consisted of interview initiation, question design, data collection, data analysis, theory establishment, and forming conclusions. Questions about travel preferences, travel activity intensity, virtual travel intentions, perceptions in the microclimate cabin, and experimental suggestions were asked to the 60 respondents who had previously participated in the experiment. The respondents were sequentially coded as W1–W60.

Thematic analysis was used to analyze the qualitative data (Braun and Clarke, 2006). The authors identified the key content from interviewees’ narratives, and it was found that the conclusions obtained from the interview data were consistent with the results in the experiment. The specific interview results are as follows.

The interview analysis showed that 56 of the 60 respondents were very interested in the temperature virtual tourism activity, 35 respondents indicated that the virtual tourism experience was similar to real travel, and they felt different levels of thermal comfort under the three types of activity states. Eighteen respondents thought that the activity intensity in virtual tourism can affect their perceptions of temperature, and 52 respondents were willing to participate in more realistic temperature virtual tourism activities in the future. Some respondents commented as follows: “I feel that the temperature virtual tourism experience is very realistic; it seems like I was in a real travel environment”; “It’s fun to watch the travel scenes while feeling the temperature changes and exercising.” Nevertheless, others showed their dislike of the temperature virtual travel experience: “The activity intensity is not accurate and not realistic enough”; “I would become distracted easily if the time was long.” Based on this, respondents put forward three suggestions: improving the simulation degree of the virtual tourism environment and temperature, adding a more realistic tourism experience and enriching the visual and auditory feedback content, and temperature virtual tourism should be more intelligent and life-oriented, being closer to the real travel experience.

It can be seen from the interview data that respondents’ virtual tourism experience could be improved by adjusting the perceived temperature under different activity intensities and simulating the virtual tourism situation to the maximum extent, thereby stimulating their interest and enthusiasm for the travel experience. The study also found that the respondents hope to interact with objects in the virtual tourism world naturally through necessary equipment, and to experience a more realistic virtual tourism environment that integrates visual, auditory, and tactile sensations. Therefore, exploring the combination of temperature, posture, sound and eye tracking, and other new interactive methods could provide a diversified and satisfying immersive interactive experience for virtual tourists.

## Conclusion

### Conclusion and Discussion

Previous studies have shown that under different temperatures, tourists perceive different microclimate comfort levels and their recreational behaviors also differ (Chen and Dong, 2015). Microclimate simulation experiments are popular in environmental psychology research, but few studies have introduced these to virtual tourism, and there has not yet been research on thermal comfort in the context of virtual tourism. The current study contributes to fill this gap. The results of this study demonstrated the relationships between environmental temperature, activity intensity, and perceived and physiological thermal comfort in the context of virtual tourism by using experimental and interview methods. The study aimed to understand the role of environmental temperature in how virtual tourists assess their thermal comfort under three types of activity intensities (sitting, slow walking, brisk walking). The findings indicated the following.

First of all, the environmental temperature affected the perceived thermal comfort of virtual tourists, and there was a significant relationship between perceived thermal comfort and environmental temperature in the virtual tourism experience. Activity intensity can moderate the relationship between environmental temperature and perceived thermal comfort, which is consistent with the findings of an existing study (Qiu et al., 2005). The current study confirmed that activity intensity did not directly affect perceived thermal comfort, but instead influenced tourists’ thermal comfort by moderating the relation between temperature and thermal comfort. Under the same activity intensity, as the temperature increased, the comfort level of tourists first increased and then decreased; under different activity intensities, the thermal comfort level of tourists also differed within the same temperature range. In addition, the results indicated that tourists’ comfortable temperature range for all activity types was 19.8–25.0°C, which is within the most acceptable outdoor environmental temperature range of 19.6–29.5°C (Shang et al., 2020).

Second, under different intensities of virtual tourism activity, the environmental temperature will affect the physiological thermal comfort of tourists. Both the tourism environment and tourists’ self-adaption will lead to changes in physiological comfort indicators (Ji et al., 2015). Therefore, it is necessary to discuss the mechanism of thermal comfort in virtual tourism from a physiological perspective. Physiological indicators provide an objective evaluation of thermal comfort and the basis for subjective thermal comfort. The study introduced activity intensity into the model and discussed the influence relationship under different activity states. From the results of covariance analysis, it can be seen that in the sitting state, the environmental temperature did not significantly affect virtual tourists’ physiological indicators. In both slow and brisk walking states, the environmental temperature had a significant effect on tourists’ blood oxygen (SPO_2_). In addition, the environmental temperature can also affect tourists’ PR significantly in the slow walking state. This study responds to the initiatives of Tussyadiah et al. (2018), who called to “include objective measurements like using sensors and psychophysiological analysis in the virtual tourism study.”

Furthermore, this research evaluated thermal comfort through both subjective and objective procedures in the microclimate simulation experiment, enriching the analysis method and expanding the studies in the relevant field (de Dear et al., 2013; Zare et al., 2018; Bogicevic et al., 2019).

### Theoretical and Practical Implications

This study has shown that the experimental method is applicable to research on virtual tourism thermal comfort to a certain extent, and this study may be one of only a few attempts to empirically assess the impacts of environmental temperature and activity intensity on tourist thermal comfort in a virtual tourism context. The current study contributes to the extension of relevant studies in two ways. First, we added analysis of tourist thermal comfort to virtual tourism research, which addressed the call to further broaden and extend virtual tourism research studies (Bogicevic et al., 2019). Previous studies mainly explored the concept and prospects of virtual tourism (Guttentag, 2010), and conducted empirical studies to assess whether or not tourists supported virtual tourism experiences (Tavakoli and Mura, 2015; Bogicevic et al., 2019). The current study analyzed virtual tourism experiences in different activity intensities, which is quite rare in previous studies.

Second, this study employed a microclimate simulation experiment in the context of virtual tourism. Tourism microclimate simulation experiments are mostly used to study tourist perceptions and behaviors in specific tourist attractions (Galagoda et al., 2018). The current study combined physiological parameters and a microclimate simulation experiment within virtual tourism research, conducting a fine-grained analysis of the influence of temperature on the comfort of tourists. It provides a new perspective to explore the virtual tourism space situation and enriches the content of virtual tourism (Tussyadiah et al., 2018).

From a practical standpoint, the findings also provide significant implications for tourism managers. First, in this study, we have demonstrated that environmental temperature was significantly related not only to tourists’ perceived thermal comfort but that it also contributed to their physiological indicators of thermal comfort. These findings extend our understanding of tourists’ virtual tourism experience and offer a novel view on providing a successful service to virtual tourists. Thus, destination managers should utilize temperature to improve the immersion and satisfaction of the virtual tourism experience, which are the biggest drawbacks of virtual tourism (Cheong, 1995). Tourists’ psychological and physiological responses result from interaction between tourists and the travel time and space. The simulation of the travel situation from the aspects of climate environment, activity scenes, and visual effects can improve the simulation degree of virtual tourism, enhancing the sense of interaction and immersion of the experiment.

Second, we have also confirmed the moderating effect of activity intensities on the relationship between ambient temperature and thermal comfort. Therefore, future construction of virtual tourism scenes should control tourists’ activity intensity and help to relieve visitors’ fatigue. Based on the theoretical evidence of the human–environment relation, through the creation and design of virtual tourism scenarios, realizing the control and design of tourists’ activity intensity helps to provide a better experience.

Third, as suggested by our results, high levels of realistic visual, audio, and tactile sensations in virtual tourism promoted tourists’ positive perceptions and satisfaction. Thus, tourism managers should recognize the development trend and important role of virtual tourism in the post-COVID-19 period (in non-crisis situations). Virtual tourism is not a comprehensive replacement of the traditional tourism industry, but assists in promoting the sustainability of tourism destination brands (Rácz and Zilizi, 2019). Tourism destinations may consider designing and developing virtual tourism programs based on the environmental temperature. Through a reasonable combination of virtual tourism and environmental temperature, the thermal comfort of tourists can be promoted, and the difference between low and peak travel seasons can be reduced.

### Limitations and Future Research

Like other research studies, this study also has limitations. First, future studies could try to improve tourists’ sense of immersion in virtual tourism. The tourism experience is an interactive process between tourists and travel time and space. This study uses videos to replace the virtual tourism experience, which is not accurate enough. Future research could combine AR, VR, MR, and other equipment to improve the simulation degree of virtual tourism and construct a more realistic tourism scenario, resulting in a more rigorous experiment. Second, this study only considers thermal comfort as the outcome; human body comfort evaluation is a psychological state of feeling satisfied with the environmental temperature. Future research studies could integrate tourism fatigue and time sensation in the model to conduct a comprehensive tourism experiment. Third, the temperature intervals selected in the study were mainly in a milder range, and most of the respondents were young. Future researchers could conduct comparative experiments based on age, physical condition, and region to obtain more extensive and universal results.

## Data Availability Statement

The raw data supporting the conclusions of this article will be made available by the authors, without undue reservation.

## Ethics Statement

The studies involving human participants were reviewed and approved by School of Management, Shandong University. The patients/participants provided their written informed consent to participate in this study. Written informed consent was obtained from the individual(s) for the publication of any potentially identifiable images or data included in this article.

## Author Contributions

All authors listed have made a substantial, direct, and intellectual contribution to the work, and approved it for publication.

## Conflict of Interest

HC was employed by the company Jinan Lixia Holding Group Co., Ltd. The remaining authors declare that the research was conducted in the absence of any commercial or financial relationships that could be construed as a potential conflict of interest.

## Publisher’s Note

All claims expressed in this article are solely those of the authors and do not necessarily represent those of their affiliated organizations, or those of the publisher, the editors and the reviewers. Any product that may be evaluated in this article, or claim that may be made by its manufacturer, is not guaranteed or endorsed by the publisher.
